# Non-adherence to antipsychotic medication, relapse and rehospitalisation in recent-onset schizophrenia

**DOI:** 10.1186/1471-244X-8-32

**Published:** 2008-04-30

**Authors:** Gunnar Morken, Jan H Widen, Rolf W Grawe

**Affiliations:** 1 Department of Neuroscience, Faculty of Medicine, NTNU, N-7006 Trondheim, Norway; 2 Division of Psychiatry, ∅stmarka, St. Olavs University Hospital, N-7006 Trondheim, Norway; 3 Clinic of Psychiatry, Telemark Hospital, Skien, Norway; 4 SINTEF Health Research, Trondheim, Norway

## Abstract

**Background:**

The aims of this study were to describe outcome with respect to persistent psychotic symptoms, relapse of positive symptoms, hospital admissions, and application of treatment by coercion among patients with recent onset schizophrenia being adherent and non-adherent to anti-psychotic medication.

**Materials and methods:**

The study included 50 patients with recent onset schizophrenia, schizoaffective or schizophreniform disorders. The patients were clinically stable at study entry and had less than 2 years duration of psychotic symptoms. Good adherence to antipsychotic medication was defined as less than one month without medication. Outcomes for poor and good adherence were compared over a 24-month follow-up period.

**Results:**

The Odds Ratio (OR) of having a psychotic relapse was 10.27 and the OR of being admitted to hospital was 4.00 among non-adherent patients. Use of depot-antipsychotics were associated with relapses (OR = 6.44).

**Conclusion:**

Non-adherence was associated with relapse, hospital admission and having persistent psychotic symptoms. Interventions to increase adherence are needed.

**Trial registration:**

Current Controlled Trials NCT00184509. Key words: Adherence, schizophrenia, antipsychotic medication, admittances, relapse.

## Background

Antipsychotic medications have reduced the number of recurrent psychotic episodes among persons with schizophrenia [1]. Poor adherence to antipsychotic medication in schizophrenia have been associated with rehospitalisation [2,3] and relapse [4] in cross sectional studies and studies monitoring hospital admissions among patients being in shortage of supplies of antipsychotic medications [5]. Data from the recent Clinical Antipsychotic Trials of Intervention of Effectiveness (CATIE) study revealed that 74 % of the patients discontinued their medication before 18 months, i.e. either to non-adherence or to another antipsychotic medication [6]. There is, however, a paucity of prospective studies that in detail compare the development of symptoms and disability among medication adherent and non-adherent patients with recent onset schizophrenia. Robinson [7] demonstrated a high frequency of rehospitalisations in a group of patients with schizophrenia containing both non-adherent patients and patients with a planned discontinuation of antipsychotic medication. Non – adherent patients might have different clinical characterisations than patients with planned discontinuation of their medication due to a long time without alarming symptoms.

The present report is a part of a randomized controlled trial of two years continued integrated treatment compared with standard treatment in recent-onset schizophrenia [8,9] [see Additional file 1, 2, 3]. The two interventions seemed to be equal in effects on adherence to medication and the proportions of patients that were adherent to medication throughout the study were much the same in the integrated treatment and the standard treatment groups. The aims of the present report were to compare outcome with respect to persistent psychotic symptoms, relapse of positive symptoms, hospital admissions and application of treatment by coercion among patients with recent onset schizophrenia being adherent and non-adherent to anti-psychotic medication.

## Materials and Methods

### Sample

Consecutive patients referred to a specialized psychiatric team for psychosis were asked to participate in the study. The catchment area of the team was the northeast half of Sør-Trøndelag County in the middle of Norway during the first 2 years of the study and the entire county for the rest of the inclusion period. The whole county has a population of about 270.000 inhabitants. The recent-onset patients were selected for the study if they were diagnosed with DSM-IV [10] schizophrenia, schizoaffective or schizophreniform disorders by psychologists or psychiatrists trained to administer the Structured Clinical Interview for the DSM-IV [11] reliably. Clinically stable patients aged between 18 and 35 years who were prescribed antipsychotic medication and expected to reside in the county for at least 1 year after inclusion were asked to participate in the study. Patients with major substance use disorders or mental retardation were excluded. Recent-onset was defined as the emergence of distinct initial psychotic symptoms for the first time within the past 2 years. Very brief and transient experiences of psychotic symptoms prior to the past 2 years were not classified as distinct psychotic symptoms. Efforts were made to get referrals of all patients with a recent onset psychotic disorder in the catchment area. Invitations for study referral were sent to the psychiatric inpatient units, outpatient clinics, and general practitioners in the catchment area. Written informed consent was obtained after the procedures were fully explained to the patients and baseline assessments completed before inclusion from all included patients. The study was approved by The Regional Committee for Research Ethics, Middle Norway.

### Interventions

All the 50 included patients received standard treatment which was regular case management with antipsychotic drugs, supportive housing and day care, crisis inpatient treatment at 1 of 2 psychiatric hospitals, rehabilitation that promoted independent living and work activity, brief psychoeducation, and supportive psychotherapy. In addition, the patients randomized to integrated treatment received structured family psychoeducation, cognitive – behavioural family communication and problem solving skills training, intensive crisis management provided at home, and individual cognitive-behavioural strategies for residual symptoms and disability [12,13]. More details of the intervention and the results on new episodes, rehospitalisation, function and adherence to antipsychotic medication are reported elsewhere [8,9]. The two interventions seemed to be equal in effects on adherence to medication. The dose of antipsychotic medication was kept to the lowest effective level. Although combination therapy and switch of antipsychotics occurred, monotherapy was preferred and plasma assays were used on clinical indications to optimise dose and to verify adherence. The physicians had no restrictions when they selected antipsychotics. Some of the patients having problems adhering to oral medication were offered depot injections.

### Assessments

Registration of antipsychotic medication adherence was based on patient interviews. Information on adherence was also gathered from therapists, carers, plasma assays, and patient records. The information on adherence gathered from plasma assays was not assessed on a regular basis. If a patient later revealed that she or he had given incorrect information about adherence at an earlier visit, the records for adherence were corrected. Medication adherence was recorded at inclusion and bimonthly for the 2 years, and information gathered at later visits and information from informal carers, therapists and patient records was used to compensate for missing values. Adherence was graded on a 4 point scale according to Tarrier [14]: 0: Up to 1 week without medication, 1: Up to 1 month without medication or 4 times more than 1 week without medication, 2: Up to 5 months without medication, 3: five months or more without medication. Patients with 1 month or more or 4 single weeks or more without medication were rated as non-adherent with medication. Patients receiving depot injections of antipsychotics at any time were recorded as depot users. Because use of depot antipsychotics was nearly always associated with non-adherence with oral antipsychotics, adherence with oral antipsychotics was also compared with non-adherent patients and the depot users combined. Target Psychotic Symptoms measured each individual's unique hallucinations, delusions and thought disorders on a 0–7 scale [15]. Suggesting symptom exacebarations, extra measure times were executed. Brief Psychiatric Rating Scale, BPRS, was assessed bi-monthly [16]. The Positive and Negative Symptom factors derived for first episode schizophrenia were used to measure these dimensions [17].

Global Assessment of Functioning; GAF [10], assessed overall functioning at 0, 12 and 24 months.

Continuous records were kept of medication and psychosocial treatment adherence, hospital admissions and suicidal behaviour.

Ratings were made by independent raters who were blind to treatment conditions and trained to obtain a 0.8 kappa coefficient of inter-rater reliability on all rating scales.

### Operationalised variables

Psychotic recurrences and exacerbations: An episode of relapse was defined as a two-point increase and a score of six or seven on the Target Symptoms Ratings Scale (0–7) AND a score of six or seven on one of the key psychotic symptom items on the BPRS (1–7). In addition this was confirmed by an independent person (researcher, family member, clinician, case manager, etc) as a significant worsening [18]. Persistent psychotic symptoms were defined as scoring more than four on BPRS hallucinations or unusual thought content for more than six consecutive months during the study period. Symptoms at baseline were assessed by the Brief Psychiatric Rating Scale (BPRS), Target Symptoms Rating Scale and Global Assessment of Function (GAF). Substance abuse was not systematically assessed after the patients were included in the study. Records were kept of medication and psychosocial treatment adherence, hospital admissions and suicidal behaviour. All included patients were included in the outcome analyses.

### Statistics

Categorical factors were compared using Chi Square tests. Ordinal variables were compared using the Mann-Whitney test. To predict outcome, i.e. persistent psychosis, relapse and admittances to hospital, separate logistic regression analyses were performed with adherence to antipsychotic medication or use of depot antipsychotics as the explanation variable. All data analyses were performed with the Statistical Package for Social Sciences Version 14.0. An alfa level of 0.05 was selected.

## Results

Of 168 consecutive referrals, 96 patients met the criteria for schizophrenic disorders. Forty six patients were excluded; 21 were not recent onset, 4 had substance abuse, 4 lived outside of the catchment area, 4 would not give written consent, 2 had mental retardation and 11 did not recover from the initial psychotic episode. One of the included patients moved to another region and 6 patients received limited psychosocial treatment and follow-up. However, it was possible to reach all patients for evaluations. Demographic and clinical characteristics at baseline are shown in Table 1. The 19 women and 31 men who were included in the study came from hospital wards (57 %), outpatient clinics (23 %) and general practitioners (21 %). Patients were clinically stable at baseline and were assumed to be adherent with medication. At the end of the study, 5 (10 %) patients revealed that they did not take their medication as prescribed at baseline. All the patients had been prescribed antipsychotic medication. During the 2 years, 37/50 patients were given first generation antipsychotics, 12/50 patients were given second generation antipsychotics excluding clozapine and 16/50 were given clozapine.

**Table 1:** Baseline characteristics of the 50 included patients.

Age in years (SD)	25.4 (4.6)
Female	19
Male	31
Hospitalised before study	42
Diagnosis	
Schizophrenia	40
Schizoaffective	6
Schizophreniform	4
Mean BPRS score (SD)	40 (7.6)
Mean GAF score (S.D.)	50 (10.6)
Contact with family No (%)	
Living with parents/family	28 (56)
In weekly contact with parents/family	14 (28)
None or little contact	8 (16)
Days hospitalised last 12 months before study, mean (SD)	124 (105)

Good adherence was found in 19 of 29 patients that anytime during the trial received oral first generation antipsychotics, 10 of 12 patients that anytime during the trial received second generation antipsychotics and 12 of 16 patients receiving clozapine (ChiSqu = 1.44 df = 2, p = 0.5). Among patients with only one medication during the whole trial, good adherence was found in 11 of 17 receiving oral first generation antipsychotics, 3 of 4 receiving second generation antipsychotics and 5 of 7 receiving clozapine (ChiSqu = 0.21 df = 2, p = 0.9). Patients with good adherence with oral or depot antipsychotic medication combined were more seldom persistent psychotic, had fewer relapses and admittances to hospital, fewer days in hospital and fewer days in-or outpatient admittance by coercion than patients non-adherent to antipsychotics (Table 2). Ten of 20 patients admitted to hospital were non-adherent to oral and depot antipsychotic medication combined while 6 of 30 patients not admitted to hospital were non-adherent to oral and depot antipsychotic medication combined. Eleven of 17 patients with a major recurrence were non-adherent while 5 of 33 patients without a major recurrence were non-adherent to oral or depot antipsychotic medication combined (Table 2). Patients with good adherence to oral antipsychotics compared to non-adherent patients and users of depot antipsychotics combined were more seldom persistent psychotic, had fewer relapses, a higher GAF at 2 years and better improvement in GAF during the 2 years (Table 2). Users of depot antipsychotics had more major relapses, higher number of hospitalisations, more days admitted by coercion as in-and outpatients and a lesser improvement in GAF through the 2 years compared to patients that not used depot antipsychotics (Table 2). Among the 22 patients that were non-adherent to oral antipsychotics, 4 of the 6 women and 7 of the 16 men were admitted to hospital during the 2 years. The non-adherent women had more days outpatient treatment by coercion (p = 0.010, Mann-Whitney test) and more days inpatient treatment by coercion (p = 0.049, Mann-Whitney test) than the non-adherent men (Table 3).

**Table 2:** Adherence to antipsychotic medication, use of depot antipsychotics, psychotic symptoms, relapse and admittances to hospital through two years in recent onset schizophrenia.

	Adherence with oral or depot antipsychotics				Adherence with oral antipsychotics				Use of depot antipsychotics			
			
	Good N = 34	Not good N = 16	Chi Squ	P	Good N = 28	Not good N = 22	Chi Squ	P	Not use N = 38	Use N = 12	Chi Squ	P
Persistent psychotic	6 (18%)	7 (44%)	3.85	.050	4 (14%)	9 (41%)	4.54	.033	8 (21%)	5 (42%)	2.01	.156
Relapse	6 (18%)	11 (69%)	12.66	.000	3 (11%)	14 (64%)	15.38	.000	9 (24%)	8 (67%)	7.51	.006
Admitted to hospital	10 (29%)	10 (63%)	4.96	.026	9 (32%)	11 (50%)	1.64	.201	13 (34%)	7 (58%)	2.21	.137

	Median (Range)	Median (Range)		P	Median (Range)	Median (Range)		P	Median (Range)	Median (Range)		P

Days in hospital	0 (0–295)	12 (0–264)		.038	0 (0–295)	1 (0–264)		.242	0 (0–295)	12 (0–264)		.133
Nb of hospitalisations	0 (0–7)	1 (0–16)		.011	0 (0–4)	1 (0–16)		.074	0 (0–4)	1 (0–16)		.048
Days inpatient coercion	0 (0–235)	0 (0–332)		.004	0 (0–235)	0 (0–332)		.013	0 (0–332)	10 (0–262)		.008
Days outpatient coercion	0 (0–514)	0 (0–365)		.049	0 (0–17)	0 (0–514)		.006	0 (0–360)	0 (0–514)		.004
GAF two years	59 (15–91)	48 (32–85)		.116	64 (34–91)	48 (15–85)		.014	61 (32–91)	50 (15–65)		.065
GAF change	7 (-19–16)	2 (-24–18)		.154	9 (-15–45)	0 (-20–33)		.008	8 (-20–45)	0 (-18–15)		.040

**Table 3:** Sex differences in adherence to antipsychotic medication, use of depot antipsychotics, psychotic symptoms, relapse and admittances to hospital through two years in recent onset schizophrenia

	Adherence with oral or depot antipsychotics				Adherence with oral antipsychotics				Use of depot antipsychotics		
			
	Good	Not good	Chi Squ	P	Good	Not good	Chi Squ	P	Not use	Use	P
Men N = 31	N = 18	N = 13			N = 15	N = 16			N = 24	N = 7	
Persistent psychotic	2 (11%)	7 (54%)	6,69	,010	1 (7%)	8 (50%)	7,06	,008	5 (21%)	4 (57%)	,15 F
Relapse	3 (17%)	8 (62%)	6,64	,010	2 (13%)	9 (56%)	6,23	,013	7 (29%)	4 (57%)	,21 F
Admitted to hospital	4 (22%)	7 (54%)	3,30	,069	4 (27%)	7 (44%)	0,99	,320	7 (29%)	4 (57%)	,21 F

Women N = 19	N = 16	N = 3			N = 13	N = 6			N = 14	N = 5	
Persistent psychotic	4 (25%)	0 (0%)		1,0 F	3 (23%)	1 (17%)		1,0 F	3 (21%)	1 (20%)	1,0 F
Relapse	3 (19%)	3 (100%)		,021 F	1 (8%)	5 (83%)		,003 F	2 (14%)	4 (80%)	,017 F
Admitted to hospital	6 (38%)	3 (100%)		,087 F	5 (38%)	4 (67%)		,350 F	6 (43%)	3 (60 %)	,628 F

F = Fischers exact test

Among the 12 patients receiving depot antipsychotics, 8 were treated with depot when included in the study. Of these 8, 2 used depot the whole period with good adherence, 2 had good adherence to depot, changed to oral antipsychotics with bad adherence and 4 had good adherence to depot, changed to oral with good adherence. Four patients used oral antipsychotics when included, had bad adherence and changed to depot antipsychotics with good adherence.

This means that among the 12 patients treated with depot antipsychotics some time during the study, 6 were adherent throughout the 2 years, while 6 were non-adherent. Five of 6 depot users in the non-adherent group had a relapse compared to 3 of 6 in the adherent group. One of 6 depot users in the adherent group and all 6 depot users in the non-adherent group were hospitalised (p = 0.015, Fischer Exact test) (Table 4).

**Table 4:** Odds for being persistent psychotic, having a relapse or being admitted to hospital for patients adherent or non adherent to antipsychotics or depot users.

	β coefifisient (SE)	Wald	p	Unadjusted Odds Ratio (95% CI)
Adherence with oral or depot antipsychotics				
Persistent psychotic	1.29 (.68)	3.64	.056	3.63 (.967–13.64)
Relapse	2.33 (.70)	10.99	.001	10.27 (2.59–40.67)
Admitted to hospital	1.39 (.64)	4.71	.030	4.00 (1.14–13.99)

Adherence with oral antipsychotics				
Persistent psychotic	1.42 (.69)	4.23	.040	4.15 (1.07–16.14)
Relapse	2.69 (.76)	12.61	.000	14.58 (3.32–64.03)
Admitted to hospital	.75 (.59)	1.62	.204	2.11 (.67–6.68)

Use of depot antipsychotics				
Persistent psychotic	.99 (.71)	1.94	.16	2.68 (.67–10.73)
Relapse	1.96 (.72)	6.67	.010	6.44 (1.57–26.51)
Admitted to hospital	.99 (.68)	2.13	.14	2.69 (.71–10.17)

## Discussion

### Consequences of non adherence to antipsychotic medication

Non-adherence with oral or depot antipsychotic medication combined were associated with increased frequencies of relapses, being persistent psychotic and an increased risk of being admitted to hospital in this group of patients with recent onset schizophrenia. This is in accordance with earlier reports of patients being in shortage of antipsychotic medication being frequently admitted to hospital [19].

Also measured in total number of days in hospital, in number of admittances, number of days inpatient and outpatient treatment by coercion, the patients non-adherent to oral or depot antipsychotic medication had less favourable outcomes than the adherent group. Register based studies describe much the same tendency [5,19]. The relapse rate after discontinuation of antipsychotic medication is believed to be more than 50% [20,21] and more than 50% of patients readmitted to hospital have discontinued their medication [22,23]. Even in the present study where the authors assume 90 % of the patients were adherent to medication at inclusion, 50 % of the patients admitted to hospital during 2 years were non – adherent to antipsychotic medication. Non-adherence to antipsychotic medication was associated with an Odds Ratio of 10.27 of having a major psychotic relapse. Robinson [7] demonstrated a high frequency of rehospitalisations in a group of patients with schizophrenia containing both non-adherent patients and patients with a planned discontinuation of antipsychotic medication. The patients that planned, together with their physician, to discontinuate their antipsychotic medication might have a much more benign illness than non-adherent patients included in the same discontinuation group. Days admitted by coercion as inpatients and as outpatients were more frequent among non-adherent patients in the present study. There is a hope that improved adherence to antipsychotic medication might make patients with schizophrenia more likely to be treated without coercion. Patients with good adherence to oral antipsychotics had a higher GAF level at 2 years and some improvement in GAF level during the 2 years compared to non-adherent patients including the depot users. This better outcome among patients adherent to oral antipsychotic medication might be interpreted as a result of the continuous availability of medication, but there is also the possibility that it is easier for patients with a good function and low level of symptoms to take their medication on a daily basis. The association between poor outcome, increased symptoms, low function and non-adherence is by most authors believed to be a causative relationship; i.e. nonadherence is leading to poor outcome. There is also a possible contribution to the poor outcome that non-adherent patients might have a more malignant course of the illness both leading to non-adherence and poor outcome.

### Users of depot-antipsychotics

The patients using depot-antipsychotic medication had more relapses and more days treated by coercion both as inpatients and outpatients and less improvement in GAF-score during 2 years than the rest of the patients indicating that depot-users of antipsychotics are patients with more symptoms, lower function and less ability to cooperate with the health services than patients with schizophrenia that do not use depot antipsychotics. Even if 8 of 12 users of depot-antipsychotics had relapses, only 1 of 6 adherent depot-users was admitted to hospital compared to 5 of 6 non-adherent users of depot antipsychotics. This indicates that depot-users in general have a more malignant illness than the rest of the patients, but that the depot antipsychotic medication might help the patients not to be admitted to hospital. A comparison of adherence to oral antipsychotics with non-adherence patients combined with depot users might be problematic. Some patients may use depot antipsychotics on other indications than non-adherence with oral antipsychotics. At inclusion, all the patients in the present study were in a relative stable clinical condition. The clinicians and raters believed that all the patients took their medication as prescribed at baseline, and with the exception of the 5 (10%) patients who later revealed that they had not taken their medication at baseline, we have taken this for granted. Measurement of adherence in the present study was mainly based on patient interviews. It is a problem both for patients, their families and physicians to accurately identify adherence. Patients with substance misuse or dependency, patients refusing to participate in the trial, patients not discharged from their inpatient status and patients having been ill for more than two years were excluded. Thus we may have included a group of patients often believed to be the most adherent to treatment of patients suffering from schizophrenia. The present study has some limitations: Patients with major substance use disorders or mental retardation were excluded from the study. Substance abuse is a robust predictor of antipsychotic non-adherence and treatment discontinuation. The patients had to be clinically stable, prescribed antipsychotic medication and believed to be adherent to medication at baseline and adherence to medication was not systematically evaluated biologically. The study does not differentiate between first-generation and second-generation antipsychotics (excluding clozapine) and clozapine regarding the effect on drug adherence. Problems with efficacy or tolerability of antipsychotics may decrease a patient's volitional adherence and affect clinical state. The small numbers of participants, especially of those using depot-antipsychotics is also a limitation of the study.

## Conclusion

Non-adherence to antipsychotic medication was associated with psychotic relapses and admissions to hospital. Users of depot antipsychotics had an increased frequency of relapses. When users of depot antipsychotics were adherent to medication, they had a low frequency of hospital admissions. Interventions to improve adherence to antipsychotic medication in schizophrenia is emphasised.

## Competing interests

The authors declare that they have no competing interests.

## Authors' contributions

RWG and JHW designed the study, wrote the protocol and collected the data. GM undertook the statistical analysis and wrote the first draft of the manuscript. All authors contributed to and approved the final manuscript.

## Pre-publication history

The pre-publication history for this paper can be accessed here:



## Supplementary Material

Additional file 1 Earlier publications. A list of earlier publications from the same study. Click here for file

Additional file 2 Acta psych scand. Abstract of the paper presenting the results of the randomized controlled study comparing integrated treatment and standard treatment for recent onset schizophrenia. Click here for file

Additional file 3 j of clinical psychiatry1. Abstract of the paper comparing the effects on adherence to antipsychotic medication in a randomized controlled study comparing integrated treatment and standard treatment for recent onset schizophrenia. Click here for file
