# Supplementary material for: Non-adherence to antipsychotic medication, relapse and rehospitalisation in recent-onset schizophrenia
Source: BMC Psychiatry. 2008 Apr 30;8:32. doi: 10.1186/1471-244X-8-32 (PMC2390550; doi:10.1186/1471-244X-8-32)
Supplement: Additional file 1 — Earlier publications. A list of earlier publications from the same study. [file 1471-244X-8-32-S1.doc]

## Acta Psychiatrica Scandinavica

Volume 114 Issue 5 Page 328 - November 2006

Page 328–336.
doi:10.1111/j.1600-0447.2006.00799.x

# Two years of continued early treatment for recent-onset schizophrenia: a randomised controlled study

Grawe RW, Falloon IRH, Widen JH, Skogvoll E.

### Abstract

Objective: This random-controlled study evaluated benefits derived from continued integrated biomedical and psychosocial treatment for recent-onset schizophrenia.

Method: Fifty cases of schizophrenia of less than 2 years duration were allocated randomly to integrated or standard treatment (ST) for 2 years. ST comprised optimal pharmacotherapy and case management, while IT also included cognitive-behavioural family treatment, that incorporated skills training, cognitive-behavioural strategies for residual psychotic and non-psychotic problems and home-based crisis management. Psychopathology, functioning, hospitalisation and suicidal behaviours were assessed two monthly and a composite index, reflecting overall clinical outcome was derived.

Results: IC proved superior to ST in reducing negative symptoms, minor psychotic episodes and in stabilising positive symptoms, but did not reduce hospital admissions or major psychotic recurrences. The composite index showed that significantly more IC patients (53%) had excellent 2-year outcomes than ST (25%).

Conclusion: Evidence-based treatment achieves greater clinical benefits than pharmacotherapy and case management alone for recent-onset schizophrenia.

## Journal of Clinical Psychiatry

Volume 68, Issue 4, April 2007.

Page 566 - 571

# Effects of integrated treatment on medication adherence in a randomised trial in recent-onset schizophrenia.

Gunnar Morken, Rolf W. Grawe, Jan H. Widen

Previous presentation: Poster at the18th ECNP Congress October 22-26, 2005, Amsterdam, The Netherlands.

No conflicts of interest.

### Abstract

Objective: Interventions improving adherence to antipsychotic medication are needed. The present study examined the effects on medication adherence of two years of Integrated Treatment for patients with schizophrenia.

Method: Adherence to medication was examined in a randomised controlled trial of two years of Integrated Treatment (IT) versus Standard Treatment (ST). The 50 included patients were consecutively referred to a specialized psychiatric team for psychosis and were diagnosed with DSM-IV schizophrenia, schizoaffective or schizophreniform disorders. The patients were clinically stable and had less than two years duration of the illness. IT consisted of assertive outreach community treatment, family psychoeducation and involvement and social skills training. Good adherence was defined as less than one month without medication. Outcomes were compared over 12-month and 24-month follow-up periods.

Results: No difference in adherence between the IT group and the ST group (Chi Squ = 0.06, df = 1, ns) was found. Men were more non-adherent than women (OR 6.11 (CI 1.25-29.74), p = 0.025). Patients living in families with low Expressed Emotions (EE) were less adherent than patients living in families with high EE (OR 6.04 (CI 1.07-34.13), p = 0.042).

Conclusion: No effects of Integrated Treatment on medication adherence were found.
