# Supplementary material for: Non-adherence to antipsychotic medication, relapse and rehospitalisation in recent-onset schizophrenia
Source: BMC Psychiatry. 2008 Apr 30;8:32. doi: 10.1186/1471-244X-8-32 (PMC2390550; doi:10.1186/1471-244X-8-32)
Supplement: Additional file 2 — Acta psych scand. Abstract of the paper presenting the results of the randomized controlled study comparing integrated treatment and standard treatment for recent onset schizophrenia. [file 1471-244X-8-32-S2.pdf]

Acta Psychiatr Scand 2006: 114: 328–336
All rights reserved
DOI: 10.1111/j.1600-0447.2006.00799.x

Copyright © 2006 The Authors
Journal Compilation © 2006 Blackwell Munksgaard
ACTA PSYCHIATRICA
SCANDINAVICA

Two years of continued early treatment for recent-onset schizophrenia: a randomised controlled study

Grawe RW, Falloon IRH, Widen JH, Skogvoll E. Two-years of continued early treatment for recent-onset schizophrenia: a randomised controlled study.

Objective: This random-controlled study evaluated benefits derived from continued integrated biomedical and psychosocial treatment for recent-onset schizophrenia.
Method: Fifty cases of schizophrenia of less than 2 years duration were allocated randomly to integrated or standard treatment (ST) for 2 years. ST comprised optimal pharmacotherapy and case management, while IT also included cognitive-behavioural family treatment, that incorporated skills training, cognitive-behavioural strategies for residual psychotic and non-psychotic problems and home-based crisis management. Psychopathology, functioning, hospitalisation and suicidal behaviours were assessed two monthly and a composite index, reflecting overall clinical outcome was derived.
Results: IC proved superior to ST in reducing negative symptoms, minor psychotic episodes and in stabilising positive symptoms, but did not reduce hospital admissions or major psychotic recurrences. The composite index showed that significantly more IC patients (53%) had excellent 2-year outcomes than ST (25%).
Conclusion: Evidence-based treatment achieves greater clinical benefits than pharmacotherapy and case management alone for recent-onset schizophrenia.

R. W. Grawe[1], I. R. H. Falloon[2]*, J. H. Widen[3], E. Skogvoll[4]

[1]SINTEF Health Research, Trondheim, Norway, [2]Department of Psychiatry, University of Auckland, New Zealand & Department of Psychiatry, University of L'Aquila, Italy, [3]Telemark Hospital, Norway, [4]Faculty of Medicine, Norwegian University of Science and Technology, Trondheim, Norway

Key words: randomised controlled trial; early intervention; schizophrenia; clinical outcome

Rolf W. Grawe, SINTEF Helse/SINTEF Health Research, N-7465 Trondheim, Norway.
E-mail: rolf.w.grawe@sintef.no

*Deceased

Accepted for publication March 2, 2006

Significant outcomes

- Comprehensive empirically derived pharmacological and psychosocial treatment is associated with greater reductions in negative symptoms, minor psychotic episodes, and in stabilising positive symptoms than optimal pharmacotherapy and problem-oriented case management alone for patients with recent-onset schizophrenia.
- Comprehensive treatment doubled the proportion of cases with excellent two-year clinical outcomes, but 47% of cases remained in need of continuing treatment for their persisting symptoms and/or disability, or risk of recurrence.

Limitations

- Difficulties in recruiting large numbers of recent onset cases reduced the power of the study, and, therefore, the results of this study should be interpreted with caution.
- Major psychotic episodes and hospital re-admissions did not differ in the two treatment conditions.

Introduction

Early intensive treatment of initial presentations of psychosis makes good sense. Full and lasting recovery from most health problems is associated with early detection and implementation of the most effective intervention strategies. However, despite the current wave of enthusiasm for such

services in mental health, it should be noted that to date there is relatively little evidence that early detection and intervention improves the long-term outcome of schizophrenia or other psychotic disorders (1–3). Present controlled trials of early intervention have focused mainly on pharmacotherapy (4, 5) or specific psychosocial interventions over relatively brief periods (6–9). More comprehensive programs have been evaluated mainly in cohorts, often with cases limited to adolescents and young adults, (10–13). Although the results are excellent the lack of randomised controls makes interpretations hazardous. Very recently the results of a large-scale random-controlled field trial in Denmark have shown that clinical benefits after 1 year of a flexible needs-based programme are greater than standard treatment (ST). The treatment focused on home-based assertive case management integrated with pharmacotherapy, with family or individual psychoeducation, and social and problem solving skills training offered when indicated (14).

Improvements in the effectiveness of treatment in controlling florid psychotic symptoms and reducing the risks of recurrent episodes and associated social disabilities and handicaps must rank among the greatest medical achievements of the past century (15). This has been achieved in three stages. First, the provision of psychosocial rehabilitation resources has enabled even those persons most impaired by these disorders to manage a reasonable high-quality life in the community (16). Second, the effects of neuroleptic drugs have enabled most (75–80%) acute psychotic episodes to be controlled and the rate of recurrent episodes to be halved (17). Third, the addition of stress management training, involving key carers of patients, has halved again the risk of recurrent episodes (18). Finally, there is increasing evidence that residual psychotic and non-psychotic symptoms may be reduced by innovative pharmacological and psychological strategies (19). The combination of these strategies is considered the optimal clinical management for these disorders (20–23). There is preliminary evidence that remission from all clinical and social deficits may be achieved in about 40% of cases of schizophrenia after two years of treatment that integrates all these strategies (24–25).

Treatment of lower intensity may be equally effective in recent-onset cases but there is scant evidence to support this, except perhaps in treating cases that do not yet meet the syndromal diagnostic criteria (2, 26). For this reason it is concluded that the full range of comprehensive evidence-based treatment, targeted to patients individual problems and goals, should be implemented before efforts to consider minimisation of intensity and cost.

It is concluded that there is an urgent need to study the long-term effects of the current comprehensive approaches to treatment for initial presentations of schizophrenic disorders in rigorous controlled conditions.

Aims of the Study

The present study is one centre in the International Optimal Treatment multi-site project that aims to evaluate the effects of continuous implementation of evidence-based integrated biomedical and psychosocial interventions in routine services for patients with recent-onset and chronic schizophrenia.

Material and Methods

Subjects

The sample comprised all consecutive new referrals to mental health services of Sør-Trøndelag County, Norway, aged between 18 and 35 years, and were diagnosed DSM-IV schizophrenic disorders by raters trained to use SCID-IV interviews reliably. Cases who had experienced their onset of their first psychotic symptoms more than two years ago were excluded. However, a few cases had experienced more than one acute psychotic episode prior to seeking treatment. None of these earlier episodes had received any specific treatment prior to referral to our clinic. However, we prefer to characterise the sample as 'recent-onset' rather than 'first episodes'. When the project was initiated in 1992, no effort was made to reduce the delay in seeking treatment in this region. Cases with primary substance use disorders or mental retardation were excluded along with temporary residents who were not expecting to reside in the County for at least 1 year after inclusion. No case refused the initial screening and diagnostic procedures.

Written informed consent was obtained and baseline assessments completed before patients were randomly allocated to Integrated Treatment (IT), or ST, by an independent assistant with no knowledge of the referred patients. A secretary who was not part of the clinical service opened prenumbered envelopes with treatment group assignment according to random numbers provided by the central Optimal Treatment Project administration. Blocks were of variable size (8–12), stratified according to sex and with a ratio of IT to ST of 3 : 2 to ensure that the majority of cases received the experimental treatment. Case recruitment, allocation and retention are all summarised in Fig. 1.

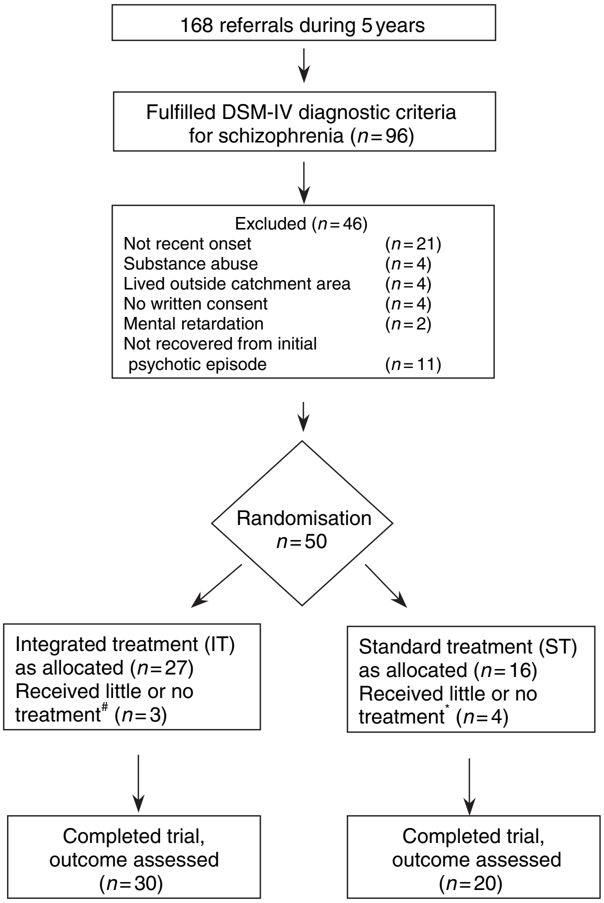

Fig. 1. Consort diagram of recruitment, allocation and retention of cases throughout 24 months.

Treatment conditions

Once acute episodes had been stabilised and patients were discharged they began treatment in the conditions to which they had been randomly assigned.

Standard treatment. ST patients received regular clinic-based case management with antipsychotic drugs, supportive housing and day care, crisis in-patient treatment at one of two psychiatric hospitals, rehabilitation that promoted independent living and work activity, brief psychoeducation, and supportive psychotherapy. 16 (80%) of the patients received ST from hospital out-patient services and the remainder from local community general health services.

Integrated treatment. Integrated Treatment (IT) Patients were treated by a multi-disiplinary team. that was independent of the ST programme. Pharmacotherapy and case management was similar to ST with a low case-load (patient-staff ratio approximately 1 : 10). In addition IT cases received structured family psychoeducation, cognitive–behavioural family communication and problem solving skills training, intensive crisis management provided at home, and individual cognitive-behavioural strategies for residual symptoms and disability. This approach is described in several published manuals (27, 28) and is almost identical to that advocated as optimal treatment for schizophrenia in recent international guidelines and reviews (20–23, 29–33). Treatment sessions were held in the home and were tailored in content and frequency to the individual goals and needs of patients and their key carers. In most cases weekly hour-long sessions were provided during the first 2 months and thereafter at least one session every third week for the first year then at least one session monthly during the second year of the project. In periods of crisis and exacerbations, intensive home-based sessions were provided up to three times a week, often supplemented with telephone consultation.

The dose of antipsychotic medication was kept to the lowest effective level taking into consideration the sensitivity of recent-onset patients to medication side effects. Monotherapy was preferred and plasma assays were frequently used to optimise dose and to check adherence. Patients, who had problems adhering to oral medication despite education and problem solving, were offered depot injections (20% in ST group, 23% in IT group).

For the 20% of the patients who had less than weekly contact with any informal carers, educational and problem solving training sessions were conducted in individual sessions.

Treatment in both conditions was goal and problem oriented and no attempt was made to match the dose of biomedical or psychosocial interventions. Regular contact between the research team and the clinical teams enabled the adherence to both treatments to be assessed. In addition to weekly case supervision, an annual review of the quality of IT treatment was conducted by an independent researcher (IRHF) (34).

Assessment

Primary outcome measures were evidence of full and stable recovery from all clinical features of schizophrenic disorders, repeated ratings of the severity of psychotic and negative symptoms and the variability of the course throughout 24 months of continuous assessment.

Target Psychotic Symptoms measured each individuals' unique hallucinations, delusions and thought disorders on a 0–7 scale every third week

throughout the study to detect psychotic exacerbations (35).

Brief Psychiatric Rating Scale (BPRS), was assessed bi-monthly (36). The Positive and Negative Symptom factors derived for first episode schizophrenia were used to measure these dimensions (37).

Global Assessment of Functioning (GAF), (38), assessed overall functioning at 0, 12 and 24 months.

Continuous records were kept of medication and psychosocial treatment adherence, hospital admissions and suicidal behaviour.

Ratings were made by an independent rater who was blind to treatment conditions and trained to obtain a 0.8 kappa coefficient of inter-rater reliability on all rating scales.

Operationalised variables. Psychotic recurrences and exacerbations: A major episode was defined as a two-point increase and a score of six or seven on the Target Symptom ratings scale (0–7) AND a score of six or seven on one of the key psychotic symptom items on the BPRS (1–7). In addition this was confirmed by an independent person (researcher, family member, clinician, case manager, etc.) as a significant worsening. A minor episode was defined in a similar way, however, the scores on Target Symptoms should be in the 4–5 range and follow a period of remission (39). Persistent psychotic symptoms were defined as scoring more than four on BPRS hallucinations or unusual thought content for more than six consecutive months during the study period.

Treatment adherence. *Good* drug adherence during 24 months was defined as no unauthorised stopping medication for more than 1 month continuously, or not discontinuing the medication on more than four occasions, each lasting at least one week. Psychosocial adherence was considered *good* if patients attended at least one session per month during each of the 24 months.

Composite clinical index (CCI). An index of good outcome throughout the 2-year period was computed. This was based on the absence of any of the following: hospital admissions; a minor or major psychotic episode; persistent psychotic symptoms; a suicidal attempt, or poor compliance with treatment.

Statistical methods

An intention-to-treat approach was used in the group outcome comparisons. Based on an earlier trial contrasting similar treatment approaches and measures over 24 months (24) it was calculated that sample sizes ranging from 24 (major recurrences) to 102 (negative symptoms) would be needed to achieve $P < 0.05$ with 80% power on the measures of target symptoms, major exacerbations, and BPRS factors. Because the control condition was considered less potent and for practical reasons an intermediate sample of 50 was considered sufficient.

Continuous and ordinal variables were reported as mean or median, with standard deviation (SD), standard error (SE), inter-quartile range (IQR), or range, as appropriate. Categorical variables were reported as percentages, and the two-tailed Fisher's exact or Pearson's chi-square tests were used for group comparisons. Simple comparisons used t-tests or Mann–Whitney's U-test for independent samples, depending on whether the assumptions of normality and homogeneity of variance were met. For continuous variables, a general linear model was used when modelling interactions and/or controlling for covariates, with repeated measures analysis of variance. The Huynh–Feldt correction was used when the assumption of sphericity was violated. The coefficient of variation was used to determine the stability of positive symptom ratings over the 13 bi-monthly BPRS assessments (24, 40). [The coefficient of variation (%) of a set of values is calculated as: 100 × (SD)/(mean value of set)].

An alpha of 0.05 was the level for significance in all analyses, and no adjustment for multiple comparisons was used unless specifically stated. The statistical software package SPSS Version 11.5 was used throughout.

Results

Of 168 consecutive referrals, 114 failed to meet the diagnostic, recent-onset or other selection criteria. Of the 54 cases that were eligible for the study, 4 refused written consent. Three of the 30 (10%) patients allocated to the IT program discontinued before 24 months (one moved to another region) and four of the 20 (20%) ST cases discontinued the two-year programme. Data were available on all these partially treated cases and none were excluded from the intention-to-treat analysis. Cases entering the study came from hospital wards (57%), out-patient clinics (23%) and general practitioners (21%).

The IT group had a significantly higher baseline GAF score than the ST group ($t = 2.3$, d.f. $= 48$, $P < 0.05$), but otherwise the study groups did not differ in any respect (see Table 1).

Three IT patients were only partial recipients of the treatment programme. One moved to another

Table 1. Demographic and clinical characteristics for the Integrated Treatment (IT) and the Standard Treatment (ST) groups

Variables	All patients ($n = 50$)	IT group ($n = 30$)	ST group ($n = 20$)
Baseline demographics			
Age of admission, mean (S.D.)	25.4 (4.6)	25 (4)	25 (4.3)
Sex, No. (%)			
Female	19 (38)	11 (37)	8 (40)
Male	31 (62)	19 (63)	12 (60)
Contact with family, No. (%)			
Living with parents/family	28 (56)	16 (53)	12 (60)
In weekly contact with family	14 (28)	9 (30)	5 (25)
None or little contact	8 (16)	5 (17)	3 (15)
Hospitalised before study entry (%)			
No	8 (16)	2 (7)	6 (30)
Yes	42 (84)	28 (93)	14 (70)
Days hospitalised last 12 months before study entry, mean (SD)	124 (105)	122.4 (105.8)	125 (105)
Psychiatric assessments, baseline			
Diagnosis (DSM-IV), No. (%)			
Schizophrenia	40 (80)	23 (76)	17 (85)
Schizoaffective	6 (12)	5 (7)	1 (5)
Schizophreniform	4 (8)	2 (17)	2 (10)
GAF score, mean (SD)	50 (10.6)	52.5 (11.2)	45.7 (8.2)
Total BPRS score, mean (SD)	40 (7.6)	38.5 (7.8)	42.8 (6.6)
Antipsychotic drugs, chlorpromazine equivalent dose per day, mean (SD)	229 (113)	208 (91)	261 (137)

region and two others refused some core aspects of the treatment. Four ST group cases received very limited psychosocial treatment and follow up. Nevertheless, major outcome assessments were completed for all patients. Missing data was less than 10% in the repeated measures, and the last observation carried forward strategy was used to replace missing assessments.

Clinical outcome (Table 2)

Half the patients in the ST group were admitted to hospital over the 2 years, compared to one-third of the IT group, with six ST (30%) and four (13%) IT cases having multiple admissions. Seventeen patients (34%) suffered a major and 16 (32%) a minor recurrence. There were significantly more minor recurrences in the ST group ($P = 0.03$). A similar proportion of cases in both conditions had persisting psychotic symptoms throughout the 2 years. There were no suicides or deaths in either group. Four IT patients and 1 ST patient made suicide attempts. The majority of cases in both conditions adhered to their treatment programmes, although significantly more IT patients (97%) complied with their psychosocial treatment than the ST group (70%). Twice as many IT patients (53%) had excellent outcomes (i.e. no recurrences, persisting psychosis, hospital admissions, suicidal behaviours or poor adherence) than ST cases (25%); this advantage was statistically significant ($\chi^2 = 4.96$ and $P < 0.05$).

Table 2. Clinical outcome in the Integrated Treatment (IT; $n = 30$) and Standard Treatment (ST; $n = 20$) groups

Variables	IT $n = 30$	ST $n = 20$	P value
Admitted to hospital (%)	10 (33)	10 (50)	ns
No. of hospital admissions (%)			
None	20 (67)	10 (50)	
One	6 (20)	4 (20)	ns
Multiple	4 (13)	6 (30)	
Major recurrence (%)	10 (33)	7 (35)	ns
Minor recurrence (%)	6 (20)	10 (50)	0.03
Minor or major recurrence (%)	14 (47)	13 (65)	ns
Persistent psychotic symptoms (%)	8 (27)	5 (25)	ns
Coefficient of variation of psychotic symptoms mean% (SD)	15.6 (10.5)	24.4 (12.7)	0.01
Suicidal behaviour (%)			
Suicide	0 (0)	0 (0)	ns
Attempt	4 (13)	1 (5)	
Good adherence to drugs (%)	20 (67)	14 (70)	ns
Good adherence to psychosocial (%)	29 (97)	14 (70)	0.01
Good outcome on Clinical Composite Index (%)	16 (53)	5 (25)	0.05

BPRS factors (see Figs 2 and 3)

Positive Symptoms were absent or minimal for most patients at baseline (mean = 11.1, SD = 3.8; on a scale with a range of 7–49), indicating that clinical stabilisation of the acute phase had been achieved successfully. There was a trend for further improvement over the 24 months assessment period ($F = 2.011$; df = 6.5, 48; $P = 0.06$ with Huynh–Feldt correction). There was no significant group by time interaction on the repeated measures analysis of variance ($F = 1.151$ df = 1, 48; $P = 0.29$). However, whereas the IT group appeared to follow a fairly stable course, ST cases seemed somewhat unstable. A t-test comparing the coefficient of variation of patients over the 13 assessments of positive symptoms supported this observation, indicating that there were significantly greater fluctuations in the levels of positive symptoms cases receiving the ST programme (24.4%) than those in IT (15.6%); $t = 3.27$, d.f. = 48, $P = 0.002$.

Negative symptoms were also relatively low at baseline (mean = 5.2, SD = 1.9; on a scale range 3–21). However, there was a significant trend for further improvement over the 24 months ($F = 3.943$; d.f. = 6.9, 48; $P = 0.000$; with Huynh–Feldt correction). The repeated measures group by time interaction was significant ($F = 8.813$, d.f. = 1.48; $P = 0.005$). Figure 3 indicates that IT cases showed greater improvement than ST. However, it may be noted that this advantage appeared from the baseline assessment. Therefore, we repeated the analysis with the baseline measure as the covariate. The result remained significant ($P = 0.01$). It was also noted (Table 1) that more patients in the IT

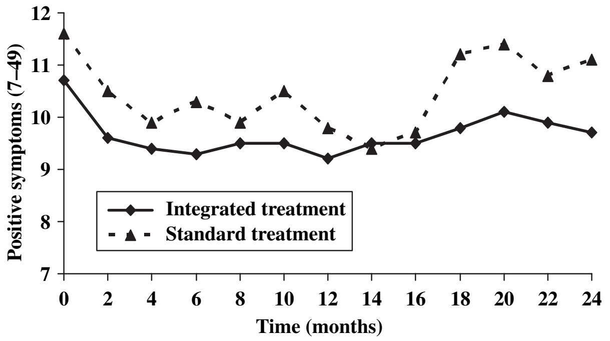

Fig. 2. BPRS positive symptoms cluster from baseline to 24 months in the two treatment conditions.

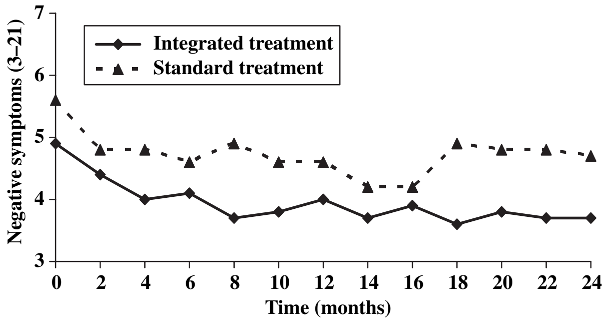

Fig. 3. BPRS negative symptom cluster from baseline to 24 months in the two treatment conditions.

group received atypical antipsychotic medications (mainly clozapine) than in the ST group. A further analysis adding the nature of pharmacotherapy as the covariate did not effect the results ($P = 0.016$).

GAF. GAF scores for the overall cohort improved from a mean of 49.8 (SD = 0.6) to 56.1 (SD = 17.2) over the 24 months. A repeated measures analysis with the initial scores (that were significantly different) as covariates showed a significant improvement over time ($F = 10.993$; d.f. = 2, 96; $P < 0.001$), but no significant group × time interaction.

Discussion

The present study suggests that integrated evidence-based pharmacological and psychosocial treatment strategies that have proven efficacy in established schizophrenic psychoses may have similar benefits in reducing clinical morbidity when applied early in the course of the disorder. Patients who received this approach over 24 months had significantly less negative symptoms and remained more stable in terms of psychotic symptoms than a cohort who received the ST programme of optimal pharmacotherapy and comprehensive case management. The proportion of cases having major episodes and hospital admissions did not differ in the two conditions, although the routine treated cases spent double the time in hospital. This lack of significant benefit was mainly accounted for by the consistently good outcome of cases receiving the ST, all of whom received some psychoeducation that has been associated with similar benefits in reducing psychotic exacerbations in first episode cases (8). The overall trend was for clinical recovery in both conditions with 25% of the ST group and 53% of the patients receiving IT having an excellent two-year clinical outcome (i.e. no persistent psychotic symptoms, psychotic exacerbations, suicides or attempts, hospital admissions, or poor adherence). This study and its results are remarkably similar to those obtained in the much larger multi-centred Danish trial (14). Both provided treatment in the home and focused on the expressed needs of patients and their families. The percentage of patients with schizophrenia who had a good outcome at 12 months (i.e. no ‘poor outcome’) were 38% in the OPUS IT cohort compared to 17% in the ST group. This represents a 21% advantage for the IT condition, compared to the 28% advantage on a similar measure in the present study. However, in our study every case was followed up every 2 months for 2 years, and those who did not adhere to the treatment programme were considered to have poor outcomes.

The 53% clinical recovery rate associated with the evidence-based treatment compared favourably with earlier studies. A 24-month study of a very similar integrated pharmacotherapy and psychosocial programme showed 40% without any positive or negative symptoms or any social disability after 2 years (24). Two-thirds of the cases in that study were of recent onset. More recent cohort studies using similar methods showed similar rates of remission from symptoms after one year (1, 10, 12). These rates of symptom remission are dramatically better than those associated with naturalistic studies where medication and supportive case management have been the basis for long-term treatment and recovery rates after 5 years for first episode cases have been between 20 and 30% (4, 5, 41–45). However, it is important to note that criteria for clinical remission and recovery in psychotic disorders have not been clearly defined, so that the terms are used in highly idiosyncratic ways that make comparisons unreliable (46, 47).

Thus, it would appear that the success of early intervention programmes depends not merely on early detection, but also on the quality of treatment

provided over the long term. Those strategies that have demonstrated their efficacy in well-controlled clinical studies should be the first line of treatment of first episode cases (1, 10, 13, 14). In this sense there would seem to be little need for specialised early intervention programmes, rather a flexible approach to treatment of schizophrenia that is focused on the current life goals and needs of individual patients and their informal carers, such as that provided in this project (27). Such an approach is highly consistent with current reviews and guidelines for optimal treatment of schizophrenic disorders (20–23, 29–33). These flexible individualised approaches that target problems rather than syndromes may prove more effective and acceptable for all patients, including those with sub-threshold symptoms or high risk factors (3, 11, 12). However, the debate as to whether specialised early intervention teams are superior to clinics for all cases of schizophrenia that are adequately staffed by professional teams trained to provide the full range evidence-based biomedical and psychosocial strategies can only be answered by empirical evidence. Such evidence is awaited eagerly.

Although the proportion of cases with benign clinical courses was doubled by evidence-based treatment, half these early cases had psychotic exacerbations and/or persisting psychosis. For these cases, 2 years of intensive treatment was insufficient and further continued intensive efforts are necessary to minimise clinical and social morbidity. Thus, early intervention must merge with continued long-term biomedical and psychosocial treatment programmes for most cases (16). The hazards of premature cessation of comprehensive biomedical and psychosocial treatments in first episode cases have been documented already (2).

It was possible that the added benefits of the IT in reduction of negative symptoms and improved stability of residual psychotic features were associated with drug adherence and pharmacotherapy factors. At the time the study started the only new antipsychotic drugs available in Norway were clozapine and risperidone. Later olanzapine was marketed. At the 24-month point 40% of the overall sample were receiving standard neuroleptics, 48% atypicals of which 30% were prescribed clozapine. The remaining five (12%) patients were withdrawn from antipsychotics ($n = 5$) or were taking only lithium ($n = 1$). More patients were prescribed first generation antipsychotics in the IT group (47%) than in the ST group (30%). While, more ST patients received clozapine (40%) than IT patients (23%). The good outcome rate in the IT group, as measured by the Clinical Composite Index, was the same with either first generation (37%) or atypical (37%) medication. By contrast none of the 11 ST cases prescribed atypical drugs had a good outcome. This suggests that, at least in the ST group, changes to the newer medicines were probably prompted by a poor clinical response (as recommended by evidence-based pharmacotherapy guidelines). This may also support the conclusion that the better outcome of negative symptoms associated with IT was unlikely to be due to the pharmacotherapy component alone.

However, it was clear that effective pharmacotherapy was an important contributor to the improved outcome of IT. Those cases that did not adhere continuously to their prescribed medication had half the rate of good outcome associated with that condition. However, the optimal pharmacotherapy of first episode cases is not well understood (48). Five of the six cases that had been withdrawn from neuroleptic drugs at 24 months showed a good outcome. Undoubtedly these were cases that showed rapid and full early recovery and were not considered likely to benefit from long-term pharmacotherapy. It is important to consider the criteria and strategies for withdrawing medication from recovered recent-onset cases. Research has focused mainly on chronic or recurrent cases and has highlighted the hazards associated with drug withdrawal, at least immediate and total withdrawal under double-blind-controlled conditions, that has often been instituted immediately upon stabilisation after an acute psychotic episode (49, 50). There is an urgent need to study withdrawal from neuroleptics in cases with full and stable remissions, particularly after a single psychotic episode (51). The 53% of cases who received IT in this study and remained free from all symptoms throughout 2 years may be considered for such a study. However, as we have noted above, a proportion of these cases may have achieved maximum benefits from continued medicines at a much earlier stage. Furthermore, it is probable that after 2 years of continued medication withdrawal might prove pharmacologically very difficult.

Acknowledgements

We acknowledge and thank: the patients and their carers; the therapists – P. J. Ingstad, T. Antonsen, A.-L. Lovaas and B. Lervag; and many colleagues who provided feedback and help at different stages - Professors K.G. Gotestam and S. Levander; and K.-H. Melle for helping us with the blind videotaped ratings. The study was funded by the Norwegian Research Council and the Norwegian Ministry of Health.

References

1. Craig TK, Garety P, Power P et al. The Lambeth Early Onset (LEO) Team: randomised controlled trial of the effectiveness of specialised care for early psychosis. BMJ 2004;**329**:1067.
2. Linszen D, Dingemans P, Lenior M. Early intervention and a five year follow up in young adults with a short duration of untreated psychosis: ethical implications. Schizophr Res 2001;**51**:55–61.
3. McGorry PD, Yung AR, Phillips LJ et al. Randomized controlled trial of interventions designed to reduce the risk of progression to first-episode psychosis in a clinical sample with subthreshold symptoms. Arch Gen Psychiat 2002;**59**:921–928.
4. Breier A, Schreiber JL, Dyer J, Pickar D. National Institute of Mental Health longitudinal study of chronic schizophrenia. Prognosis and predictors of outcome. Arch Gen Psychiat 1991;**48**:239.
5. Crow TJ, MacMillan JF, Johnson AL, Johnstone EC. The Northwick Park study of first episodes of schizophrenia. II. A randomised controlled trial of prophylactic neuroleptic treatment. Brit J Psychiat 1986;**148**:120–127.
6. Haddock G, Tarrier N, Morrison AP et al. A pilot study evaluating the effectiveness of individual in-patient cognitive-behavioural therapy in early psychosis. Soc Psych Psych Epid 1999;**34**:254–258.
7. Jackson H, McGorry P, Henry L et al. Cognitively oriented psychotherapy for early psychosis (COPE): a 1-year follow-up. Brit J Clin Psychol 2001;**40**:57–70.
8. Linszen D, Dingemans P, Van Der Does JW et al. Treatment, expressed emotion and relapse in recent onset schizophrenic disorders. Psychol Med 1996;**26**:333–342.
9. Morrison AP, French P, Walford L et al. Cognitive therapy for the prevention of psychosis in people at ultra-high risk: randomised controlled trial. Br J Psychiatry 2004;**185**:291–297.
10. Cullberg J, Levander S, Holmquist R, Mattsson M, Wieselgran I-M. One year outcome in first episode psychosis in the Swedish Parachute project. Acta Psychiatr Scand 2002;**106**:276–285.
11. Falloon IRH. Early intervention for first episodes of schizophrenia: a preliminary exploration. Psychiatry 1992;**55**: 4–15.
12. Malla AK, Norman RM, Manchanda R et al. Status of patients with first-episode psychosis after one year of phase-specific community-oriented treatment. Psychiat Serv 2002;**53**:458–63.
13. McGorry PD, Edwards J, Mihalopoulos C et al. EPPIC: An evolving system of early detection and optimal management. Schizophrenia Bull 1996;**22**:305–326.
14. Petersen L, Nordentoft M, Jeppesen P et al. Improving 1-year outcome in first epuisode psychosis. OPUS trial. Br J Psychiatry Suppl 2005;**187**:s48, 98–104.
15. Fallon IRH, Held T, Roncone R, Coverdale JH et al. Optimal treatment strategies to enhance recovery from schizophrenia. ANZ J Psychiatry 1998;**32**:43–49.
16. Kopelowicz A, Liberman RP. Integrating treatment with rehabilitation for persons with major mental illnesses. Psychiat Serv 2003;**54**:1491–1498.
17. Wyatt RJ. Neuroleptics and the natural course of schizophrenia. Schizophrenia Bull 1991;**17**:325–351.
18. Falloon IRH. Family interventions of mental disorders: efficacy and effectiveness. World Psychiat 2003;**2**:20–28.
19. Pilling S, Bebbington P, Kuipers E et al. Psychological treatments in schizophrenia: I. Meta-analysis of family intervention and cognitive behaviour therapy. Psychol Med 2002;**32**:763–82.
20. Lehman AF, Steinwachs DM. Translating research into practice. The Schizophrenia Patient Outcomes Research Team (PORT) treatment recommendations. Schizophrenia Bull 1998;**24**:1–10.
21. Lehman AF, Lieberman JA, Dixon LB et al. American Psychiatric Association; Steering Committee on Practice Guidelines. Practice guideline for the treatment of patients with schizophrenia, second edition. Am J Psychiat 2004;**161**(suppl):1–56.
22. National Institute for Clinical Excellence, NICE. Clinical Guideline 1: Schizophrenia. Core interventions in the treatment and management of schizophrenia in primary and secondary care. London: NICE, 2003.
23. Thornicroft G, Susser E. Evidence-based psychotherapeutic interventions in the community care of schizophrenia. Brit J Psychiat 2001;**178**:2–4.
24. Falloon IRH. and Associates. Family Management of Schizophrenia: Clinical, Social, Family and Economic Benefits. Baltimore: Johns Hopkins University Press, 1985.
25. Falloon IRH, Montero I, Sungur M et al. Implementation of Evidence-Based Treatment for Schizophrenic Disorders: two-year outcome of an international field trial of optimal treatment. World Psychiat 2004;**3**:104–109.
26. Falloon IRH. and The Optimal Treatment Project Collaborators. Optimal treatment for psychosis in an International Multisite Demonstration Project. Psychiat Serv 1999;**50**:615–618.
27. Falloon IRH, Fadden G. Integrated mental health care. Cambridge: Cambridge University Press, 1993.
28. Falloon IRH and O.T.P. collaborators. Integrated Mental Health Care – a Guidebook for Consumers. Perugia.: Optimal Treatment Project, ARIETE, 2001.
29. Lehman AF, Buchanan RW, Dickerson FB et al.. Evidence-based treatment for schizophrenia. Psychiatric Clinics of North America 2003;**26**:939–954.
30. American Psychiatric Association, APA. Practice Guideline for Treatment of patients with Schizophrenia. Am J Psychiat 1997;**154**(suppl. 4).
31. Bustillo J, Lauriello J, Horan W et al. The psychosocial treatment of schizophrenia: and update. Am J Psychiat 2001;**158**:163–175.
32. Mueser KT, Torrey WC, Lynde D et al. Implementing evidence-based practices for people with severe mental illness. Behav Modif 2003;**27**:387–411.
33. Kane JM, McGlashan TH. Treatment of schizophrenia. Lancet 1995;**346**:820–825.
34. Falloon IRH, Economou M, Palli A et al. A standardised measure for verifying the quality of treatment and care in mental health services: The Clinical Strategies Implementation Scale. Psychiatr Serv 2005;**56**:1584–1590.
35. Falloon IRH, Boyd JL, McGill CW et al. Family management in the prevention of exacerbations of schizophrenia: a controlled study. New Engl J Med 1982; **306**:1437–1440.
36. Ventura J, Green MF, Shaner A et al. Training and quality assurance with the Brief Psychiatric Rating Scale: 'The drift busters". Int J Meth Psych Res 1993;**3**:221–244.
37. Ventura J, Nuechterlein KH, Subotnik KL et al. Symptom dimensions in recent-onset schizophrenia and mania: a principal components analysis of the 24-item Brief Psychiatric Rating Scale. Psychiat Res 2000;**97**:129–135.
38. American Psychiatric Association. Diagnostic and Statistical Manual of Mental Disorders, 4th edn. Washington, DC: American Psychiatric Association, 1993.

39. Nuechterlein KH, Dawson ME, Gitlin M. et al. Developmental Processes in Schizophrenic Disorders: Longitudinal studies of vulnerability and Stress. Schizophrenia Bull 1992;**18**:387–425.
40. Hauschke D, Steinijans WV, Diletti E et al. Presentation of the intra-subject coefficient of variation for sample size planning in bioequivalence studies. Int J Clin Pharmacol Ther 1994;**32**:376–378.
41. Robinson D, Woerner MG, Alvir JM et al. Predictors of relapse following response from a first episode of schizophrenia or schizoaffective disorder. Arch Gen Psychiat 1999;**56**:241–247.
42. The Scottish Schizophrenia Research Group. The Scottish first episode schizophrenia study. VIII. Five-year follow-up: clinical and psychosocial findings. Brit J Psychiat 1992;**161**:496–500.
43. Shepherd M, Watt DC, Falloon IRH et al. The natural history of schizophrenia: a five-year follow-up study of outcome and prediction in a representative sample of schizophrenics. Psychological Medicine Monograph, **15**, Cambridge: Cambridge University Press, 1989.
44. Svedberg B, Mesterton A, Cullberg J. First-episode non-affective psychosis in a total urban population: a 5-year follow-up. Soc Psychiat & Psychiat Epidemiol 2001;**36**: 332–337.
45. Wiersma D, Nienhuis FJ, Slooff CJ et al. Natural course of schizophrenic disorders: a 15-year follow-up of a Dutch incidence cohort. Schizophrenia Bull 1998;**24**:75–85.
46. Resnick SG, Rosenheck RA, Lehman AF. An exploratory analysis of correlates of recovery. Psychiat Serv 2004;**55**:540–547.
47. Manchanda R, Norman R, Malla A, Harricharan R, Takhar J, Northcott S. EEG abnormalities and two year outcome in first episode psychosis. Acta Psychiat Scand 2005;**111**: 208–13.
48. Coldham EL, Addington J, Addington D. Medication adherence of individuals with a first episode of psychosis. Acta Psychiat Scand 2002;**106**:286–90.
49. Kane JM, Marder SR. Psychopharmacologic treatment of schizophrenia. Schizophrenia Bull 1993;**19**:287–302.
50. Schooler NR. Maintenance medication for schizophrenia. Schizophrenia Bull 1991;**17**:311–324.
51. Falloon IRH. Antipsychotic drugs: When and how to withdraw them? Psychosomatics and Psychotherapy, in press.
