# Supplementary material for: Non-adherence to antipsychotic medication, relapse and rehospitalisation in recent-onset schizophrenia
Source: BMC Psychiatry. 2008 Apr 30;8:32. doi: 10.1186/1471-244X-8-32 (PMC2390550; doi:10.1186/1471-244X-8-32)
Supplement: Additional file 3 — j of clinical psychiatry1. Abstract of the paper comparing the effects on adherence to antipsychotic medication in a randomized controlled study comparing integrated treatment and standard treatment for recent onset schizophrenia. [file 1471-244X-8-32-S3.pdf]

Effects of Integrated Treatment on Antipsychotic Medication Adherence in a Randomized Trial in Recent-Onset Schizophrenia

Gunnar Morken, M.D., Ph.D.; Rolf W. Grawe, Ph.D.; and Jan H. Widen, M.A.

Objective: Interventions improving adherence to antipsychotic medication are needed. The present study examined the effects on medication adherence of 2 years of integrated treatment for patients with schizophrenia.

Method: Adherence to medication was examined in a randomized controlled trial of 2 years of integrated treatment versus standard treatment. The 50 included patients were consecutively referred to a specialized psychiatric team for treatment of psychosis and were diagnosed with DSM-IV schizophrenia or schizoaffective or schizophreniform disorder. The patients were clinically stable and had less than 2 years' duration of illness. Integrated treatment consisted of assertive outreach community treatment, family psychoeducation and involvement, and social skills training. Good adherence was defined as less than 1 month without medication. Outcomes were compared over 12-month and 24-month follow-up periods. The study was conducted from February 1992 to October 1999.

Results: No difference in adherence between the integrated treatment group and the standard treatment group ($\chi^2 = 0.06$, NS) was found. Men were more nonadherent than women (OR 6.11 [CI 1.25 to 29.74], p = .025). Patients living in families with low expressed emotion were less adherent than patients living in families with high expressed emotion (OR 6.04 [CI 1.07 to 34.13], p = .042).

Conclusion: No effects of integrated treatment on medication adherence were found.

Clinical Trials Registration: ClinicalTrials.gov identifier NCT00184509.

(J Clin Psychiatry 2007;68:566–571)

Received April 16, 2006; accepted Aug. 17, 2006. *From the Division of Psychiatry, Department of Østmarka, St. Olav's University Hospital, Trondheim (all authors); Department of Neuroscience, Faculty of Medicine, Norwegian University of Science and Technology, Trondheim (Dr. Morken); SINTEF Health Research, Trondheim (Dr. Grawe); and Clinic of Psychiatry, Telemark Hospital, Skien (Mr. Widen), Norway.*

The study was funded by the Norwegian Research Council and the Norwegian Ministry of Health.

Previously presented as a poster at the 18th congress of the European College of Neuropsychopharmacology, October 22–26, 2005, Amsterdam, the Netherlands.

Dr. Morken has received grant/research support from Eli Lilly. Dr. Grawe and Mr. Widen report no additional financial or other relationship relevant to the subject of this article.

Corresponding author and reprints: Gunnar Morken, M.D., Department of Neuroscience, Faculty of Medicine, NTNU, Østmarka, Box 3008, Lade, 7441 Trondheim, Norway (e-mail: gunnar.morken@ntnu.no).

Comprehensive outreach integrated treatment seems to be effective in enhancing the long-term outcome for patients with schizophrenia, but the effects of such treatment on medication adherence are of some controversy.[1–5] This controversy concerns the question of whether the effects of integrated treatment are mediated by enhanced adherence to antipsychotic medication or are independent effects of integrated treatment. The present report is part of a randomized controlled trial of 2 years of continued integrated treatment compared with treatment as usual in recent-onset schizophrenia.[6] The aims of the present report were to evaluate the effects of integrated treatment on adherence to antipsychotic medication and to explore predictors of medication adherence.

METHOD

Sample

Consecutive patients referred to a specialized psychiatric team for treatment of psychosis were asked to participate in the trial. The catchment area of the team was the northeast region of Sør-Trøndelag County in the middle of Norway during the first 2 years of the trial and the entire county for the rest of the inclusion period. The county has a population of about 250,000 inhabitants.

Patients with recent-onset illness were selected for the study if they were diagnosed with DSM-IV[7] schizophrenia or schizoaffective or schizophreniform disorder by psychologists or psychiatrists trained to administer the

 © COPYRIGHT 2007 PHYSICIANS POSTGRADUATE PRESS, INC © COPYRIGHT 2007 PHYSICIANS POSTGRADUATE PRESS

Table 1. Baseline Characteristics of the Included Patients

Characteristic	Integrated Treatment (N = 30)	Standard Treatment (N = 20)	All (N = 50)
Age, mean (SD), y	25.4 (4.6)	24.7 (4.3)	25.1 (4.5)
Sex, N			
Female	11	8	19
Male	19	12	31
Hospitalized before study, N			
No	2	6	8
Yes	28	14	42
Diagnosis, N			
Schizophrenia	23	17	40
Schizoaffective disorder	5	1	6
Schizophreniform disorder	2	2	4
BPRS score, mean (SD)	38.5 (7.8)	42.8 (6.6)	40.2 (7.6)
Expressed emotion, N			
High	10	7	17
Low	14	9	23
No contact with family	6	4	10

Abbreviation: BPRS = Brief Psychiatric Rating Scale.

Structured Clinical Interview for DSM-IV[8] reliably. Clinically stable patients aged between 18 and 35 years who were prescribed antipsychotic medication and expected to reside in the county for at least 1 year after inclusion were asked to participate in the study. Cases with major substance use disorders or mental retardation were excluded.

Recent onset was defined as the emergence of distinct initial psychotic symptoms for the first time within the past 2 years. Very brief and transient experiences of psychotic symptoms prior to the past 2 years were not classified as distinct psychotic symptoms. Efforts were made to get referrals of all patients with a recent-onset psychotic disorder in the catchment area. Invitations for study referral were sent to the psychiatric inpatient units, outpatient clinics, and general practitioners in the catchment area.

Written informed consent was obtained after the procedures were fully explained to the patients, and baseline assessments were completed before inclusion for all included patients. The study was approved by the Regional Committee for Research Ethics, Middle Norway, and was conducted from February 1992 to October 1999.

Interventions

Standard treatment (ST) was regular case management with antipsychotic drugs, supportive housing and day care, crisis inpatient treatment at one of 2 psychiatric hospitals, rehabilitation that promoted independent living and work activity, brief psychoeducation, and supportive psychotherapy. Sixteen (80%) of the patients received ST from psychiatric outpatient services, and the remaining received it from local community general health services.

In integrated treatment (IT), patients were treated by a multidisciplinary specialized mental health team with a low caseload (patient:staff ratio of approximately 10:1). Pharmacotherapy and case management were similar to those in ST. In addition, the patients received structured family psychoeducation, social skills training (cognitive-behavioral family communication and problem-solving skills training), and individual cognitive-behavioral strategies for residual symptoms and disability.[9,10] Treatment sessions were held in the homes of the patients and were tailored in content and frequency to the individual goals and needs of patients and their key caregivers. In most cases, weekly hour-long sessions were provided during the first 2 months, and thereafter at least 1 session was provided every third week for the first year, followed by at least 1 session each month during the second year of the project. Every family received 1 or 2 hours of education in use of medication and in methods to improve medication adherence in addition to focus on adherence in problem-solving skills training and in crisis management. In periods of crisis and exacerbations, intensive home-based sessions were provided up to 3 times a week, often supplemented with telephone consultation. For the 20% of the patients who had less than weekly contact with any informal caregivers, educational and problem-solving training sessions were conducted in individual sessions. More details of the intervention and the results of primary outcomes such as new episodes, rehospitalization, and function are reported elsewhere.[6]

The dose of antipsychotic medication was kept to the lowest effective level. Although combination therapy and switch of antipsychotics occurred, monotherapy was preferred, and plasma assays were used on clinical indications to optimize dose and to verify adherence. The physicians had no restrictions when they selected antipsychotics. Patients in both groups who had problems adhering to oral medication were offered depot injections.

The 50 included patients were randomly allocated to IT or ST after baseline assessments. Randomization was performed by an independent assistant not associated with the health services who had no knowledge of the referred patients or the clinical work. Upon a phone call, the assistant successively opened pre-numbered envelopes with group assignment that had been prepared according to a random list of numbers. Blocks were of variable size (8–12 patients), stratified according to sex and with a ratio of IT to ST of 3:2 to ensure that the majority of cases received the experimental treatment. The clinical team was blind to the procedure and the size of the blocks. Demographic and clinical characteristics for the 2 groups are shown in Table 1.

Assessments

Independent raters blind to group assignment for individual patients performed the evaluations.

Registration of antipsychotic medication adherence was based on patient interviews. Information on adherence was also gathered from therapists, caregivers,

COPYRIGHT 2007 PHYSICIANS POSTGRADUATE PRESS, INC © COPYRIGHT 2007 PHYSICIANS POSTGRADUATE PRESS, INC

Figure 1. Recruitment of Patients, Allocation, and Retention of Cases Throughout 24 Months

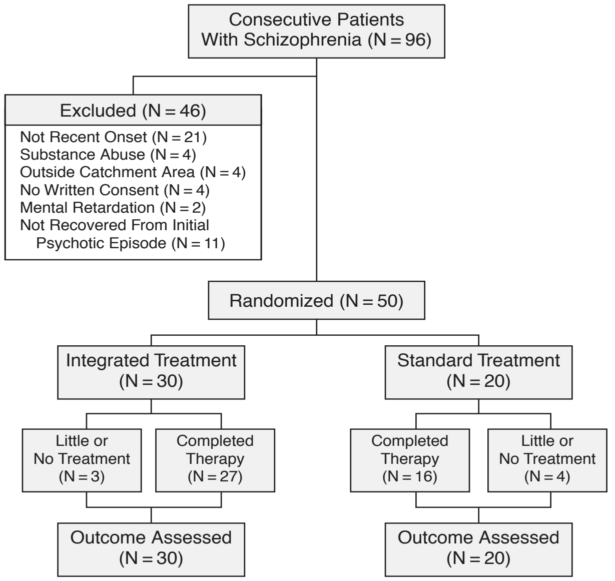

plasma assays, and patient records. If a patient later revealed that she or he had given incorrect information about adherence at an earlier visit, the records for adherence were corrected. Medication adherence was recorded at inclusion and bimonthly for the 2 years, and information gathered at later visits and information from informal caregivers, therapists, and patient records was used to compensate for missing values. Adherence was graded on a 4-point scale according to Tarrier et al.[11]: 0 = up to 1 week without medication, 1 = up to 1 month without medication or 4 times more than 1 week without medication, 2 = up to 5 months without medication, and 3 = five months or more without medication. Patients with 1 month or more or 4 single weeks or more without medication were rated as nonadherent with medication. Patients receiving depot injections of antipsychotics at any time were recorded as depot users. Because use of depot antipsychotics was nearly always associated with nonadherence with oral antipsychotics, adherence in patients taking oral antipsychotics was compared with the nonadherent patients and depot users combined. Finally, the group of nonadherent patients who did not receive any depot antipsychotics was compared to the rest of the patients.

A measure of the attitudes and feelings that a relative expressed about a mentally ill family member, termed *expressed emotion* (EE), was assessed at study entry. Expressed emotion assessments were made by a brief method known as the 5-minute speech sample, which is based upon the semistructured Camberwell Family Interview (CFI). It is derived from responses made by a patient's key relative when prompted to give thoughts and feelings about the patient for a 5-minute period. A coding system scores behaviors analogous to those rated on the CFI, such as criticism and emotional overinvolvement.[12] The 5-minute speech sample is not as sensitive in detecting high EE as the CFI, but has few false-positives.[13] Only key relatives with at least weekly contact with the patients were tested for EE.

Symptoms at inclusion were assessed by the Brief Psychiatric Rating Scale (BPRS).[14] Substance abuse was not systematically assessed after the patients were included in the study.

Records were kept of medication and psychosocial treatment adherence, hospital admissions, and suicidal behavior.

All patients were included in the outcome analyses (intention-to-treat analysis).[15]

Statistics

Categorical factors were compared using χ^2 tests. A logistic regression model was used to evaluate the relation between medication adherence at year 1 and both years combined with baseline measures and type of intervention. All data analyses were performed with the Statistical Package for Social Sciences, Version 13.0.

RESULTS

Of 168 consecutive referrals, 96 patients met the criteria for schizophrenic disorders. The progress of the subjects through the trial and reasons for exclusion are shown in Figure 1. Three patients discontinued the IT (1 moved to another region and 2 partially refused treatment). Four of the patients in the ST group received limited psychosocial treatment and follow-up. However, none were excluded from the outcome analysis (intention-to-treat analysis). The 19 women and 31 men who were included in the study came from hospital wards (57%), outpatient clinics (23%), and general practitioners (21%). Patients were clinically stable at baseline and were assumed to be adherent with medication. At the end of the study, 2 patients in the IT group and 3 patients in the ST group revealed that they did not take their medication as prescribed at baseline.

Eight of the patients had little contact with their families, and 2 patients and their families gave little information about EE and were included in the group of patients with less than weekly contact with their families. Seventeen patients were rated as having high EE, and 23 were rated as having low EE. Baseline characteristics of the patients are shown in Table 1.

All of the patients were treated with antipsychotic medication. During the 2 years, 22/30 patients in the IT group and 15/20 in the ST group ($\chi^2 = 0.17$, df = 1, p = .90) were given first-generation antipsychotics, 6/30

© COPYRIGHT 2007 PHYSICIANS POSTGRADUATE PRESS, INC © COPYRIGHT 2007 PHYSICIANS POSTGRADUATE PRESS

Table 2. Effects on Adherence to Medication of 2 Years of Integrated Treatment Versus Standard Treatment in Patients With Recent-Onset Schizophrenia[a]

	Year 1				Both Years			
Outcome	Integrated Treatment (N = 30)	Standard Treatment (N = 20)	χ^2	p	Integrated Treatment (N = 30)	Standard Treatment (N = 20)	χ^2	p
Good adherence with oral and depot antipsychotic medication	21 (70)	16 (80)	0.62	NS	20 (67)	14 (70)	0.06	NS
Good adherence with oral antipsychotics	18 (60)	13 (65)	0.13	NS	17 (57)	11 (55)	0.01	NS
Use of depot antipsychotics	7 (23)	5 (25)	0.02	NS	7 (23)	5 (25)	0.02	NS

[a]Values expressed as N (%).

Table 3. Differences in Adherence to Medication Between the Sexes and Between Families With High and Low Expressed Emotion (EE) During 2 Years[a]

	Year 1				Both Years			
Outcome	Men (N = 31)	Women (N = 19)	χ^2	p	Men (N = 31)	Women (N = 19)	χ^2	p
Good adherence with oral or depot antipsychotic medication	21 (68)	16 (84)	1.66	NS	18 (58)	16 (84)	3.70	.054
Good adherence with oral antipsychotics	18 (58)	13 (68)	0.53	NS	15 (48)	13 (68)	1.92	NS
Use of depot antipsychotics	7 (23)	5 (26)	0.09	NS	7 (23)	5 (26)	0.09	NS
	High EE (N = 17)	Low EE (N = 23)			High EE (N = 17)	Low EE (N = 23)		
Good adherence with oral or depot antipsychotic medication	16 (94)	15 (65)	4.46	.030	14 (82)	14 (61)	2.15	NS
Good adherence with oral antipsychotics	15 (88)	11 (48)	7.02	.008	13 (76)	10 (43)	4.35	.037
Use of depot antipsychotics	1 (6)	9 (39)	5.76	.016	1 (6)	9 (39)	5.76	.016

[a]Values expressed as N (%).

Table 4. Baseline Predictors of Poor Adherence to Pharmacologic Treatment During 1 Year and 2 Years in Early Intervention for Recent-Onset Schizophrenia: Results of Binary Logistic Regression Analysis (intention-to-treat analysis)

	Year 1			Both Years		
Predictor	β Coefficient (SE)	p	Odds Ratio (95% CI)	β Coefficient (SE)	p	Odds Ratio (95% CI)
Male sex	1.50 (0.86)	.082	4.46 (0.83 to 24.02)	1.81 (0.81)	.025	6.11 (1.25 to 29.74)
Standard treatment	–0.931 (0.84)	.268	0.39 (0.08 to 2.05)	–0.31 (0.72)	.670	0.74 (0.18 to 3.03)
Total BPRS score	0.12 (0.06)	.034	1.13 (1.01 to 1.27)	0.083 (0.05)	.093	1.09 (0.99 to 1.20)
Low vs high expressed emotion	2.98 (1.27)	.019	19.59 (1.64 to 234.22)	1.80 (0.88)	.042	6.04 (1.07 to 34.13)
No contact with family vs high expressed emotion	3.60 (1.44)	.012	36.43 (2.18 to 608.01)	2.01 (1.05)	.056	7.46 (0.95 to 58.58)
Constant	–9.70 (3.51)	.006		–8.02 (2.98)	.007	

Abbreviation: BPRS = Brief Psychiatric Rating Scale.

in the IT group and 6/20 in the ST group ($\chi^2 = 0.66$, df = 1, p = .42) were given second-generation antipsychotics excluding clozapine, and 8/30 in the IT group and 8/20 in the ST group ($\chi^2 = 0.98$, df = 1, p = .32) were given clozapine.

Outcome Measures

IT demonstrated no advantage over ST in terms of adherence to oral and depot antipsychotics combined, adherence to oral antipsychotics alone, or use of depot antipsychotics during either year 1 or both years (Table 2). There was a trend for women to be more adherent to medication throughout the study than men (nearly significant) (Table 3). Patients with high-EE families had better adherence to oral or depot antipsychotics in year 1, better adherence to oral antipsychotics during year 1 and in both years combined, and lesser use of depot antipsychotics than patients with low-EE families.

More men (9/31) than women (1/19) who were nonadherent to medication were not treated with depot antipsychotics through both years of treatment ($\chi^2 = 4.16$, df = 1, p = .041).

Predictors of Medication Adherence

A logistic regression model identified high total BPRS score, low EE versus high EE, and no contact with family versus high EE at baseline as predictors of poor medication adherence during year 1. Male sex and low versus high EE at baseline were identified as predictors of poor adherence during both years (Table 4). Undergoing IT

COPYRIGHT 2007 PHYSICIANS POSTGRADUATE PRESS, INC © COPYRIGHT 2007 PHYSICIANS POSTGRADUATE PRESS, INC

was not a predictor of medication adherence either at year 1 or during both years.

DISCUSSION

Integrated treatment did not increase medication adherence to either oral and depot antipsychotics combined or oral antipsychotics alone. We have previously reported better clinical outcome of IT in the same trial.[6] This earlier finding was not mediated by enhanced medication adherence and may be interpreted as the effect of the psychosocial treatment. The proportions of patients that were adherent to medication throughout the study were much the same in the IT and the ST groups. Petersen et al.,[2] in the OPUS study using much the same integrated treatment as the present study, found a reduction of positive and negative symptoms, less comorbid substance misuse, and better adherence to the treatment in general, but no significant effect on medication adherence. On the basis of a review by Zygmunt et al.,[4] our results are in accordance with most other randomized clinical trials of family interventions finding no significant effects on medication adherence, but effects on adherence to the rest of the treatment as outcome measures. In a review by Nose et al.[1] in which both medication adherence and adherence to scheduled appointments were included, positive effects of family intervention on adherence were demonstrated. It is possible that the amount of education and other interventions to improve medication adherence in the IT group in the present study was too limited to produce any group differences. It is also possible that although the patients and their families in the ST group received fewer psychosocial interventions and less support, the ST patients more often had a psychiatrist or physician as their main therapist who may have emphasized the pharmacologic treatment more than in the IT condition.

Although some results of compliance therapy for adherence to antipsychotic medication in psychotic disorders were promising,[16–19] other studies have not confirmed these findings.[20,21] Possible effective psychosocial interventions for the enhancement of medication adherence include providing behavioral components and supportive services as reminders, self-monitoring tools, and cues and reinforcements.[4]

At inclusion, all the patients in the present study were in a relatively stable clinical condition. The clinicians and raters believed that the patients took their medication as prescribed at baseline, and with the exception of the 5 patients who later revealed that they had not taken their medication at baseline, we took this for granted. The assumption of good adherence at baseline may have introduced a ceiling effect in that there was little room for improvement with therapy. Patients with substance misuse or dependency, patients refusing to participate in the trial, patients not discharged from their inpatient status, and patients who had been ill for more than 2 years were excluded from this study. Thus, we may have included a group of patients often believed to be the most adherent to treatment among patients suffering from schizophrenia. Most of the patients in the study by O'Donnell et al.[21] had been noncompliant with medication when they were included.

It is possible that effects of psychosocial interventions on medication adherence can be found in patients with poor medication adherence at inclusion.

The use of depot antipsychotics, a marker for nonadherence, was much the same in the IT and ST groups, supporting the finding of no difference in medication adherence.

When we looked at predictors at inclusion for good adherence using binary logistic regression with adherence as the dependent variable and treatment group (IT or ST), sex, total BPRS score, and high versus low EE as covariates, no effects of treatment group were revealed. Women were more adherent to medication through the 2 years of the study. This may correspond to the later onset and more benign course of schizophrenia among women. In contrast to reports of high EE as a negative factor for the course of schizophrenia, the patients living in families with high EE in the present study were more adherent to their medication than patients living in families with low EE. They were also less likely to receive depot injections of antipsychotic medication. One explanation may be that EE includes both a critical negative attitude and an overinvolvement from family members. Low EE may represent a detached emotional climate and be a marker of families who are not involved enough with the ill family member to help her or him remember to take the medication.

In a number of previous studies of EE, the 5-minute speech sample has been used as an alternative to the traditional CFI. Wearden et al.[13] conclude that the 5-minute speech sample correlates highly with the CFI in terms of classification of families as high or low EE, but tends to underrate the occurrence of high EE. More comprehensive methods of assessing EE may be needed before definitive conclusions can be made regarding this variable. Describing the effects on adherence of changes in expressed emotions during the trial was not one of the aims of the present article.

More men than women that were nonadherent to oral medication were allowed by the treatment team to be without depot antipsychotics. It is possible that the caregivers are more willing to accept that male patients with schizophrenia suffer from flourishing psychotic symptoms than to accept the same from female patients. The more benign course of the illness among women may also enhance adherence among female patients.

The relapse rate after discontinuation of antipsychotic medication is believed to be more than 50%,[22,23] and more

© COPYRIGHT 2007 PHYSICIANS POSTGRADUATE PRESS, INC © COPYRIGHT 2007 PHYSICIANS POSTGRADUATE PRESS

than 50% of patients readmitted to the hospital have discontinued their medication.[24,25] The need for interventions to improve adherence to antipsychotic medication in schizophrenia is emphasized.

CONCLUSIONS

No effects on medication adherence of IT were found. Although integrated psychosocial treatment enhances outcome for patients with schizophrenia, it does not seem to enhance medication adherence. More men than women were nonadherent to medication, and more men than women were allowed by the staff to be nonadherent without being treated with depot antipsychotics. Patients living in families with high EE were more adherent and less likely to use depot antipsychotics than patients living in low-EE families.

Drug name: clozapine (Clozaril, FazaClo, and others).

REFERENCES

1. Nose M, Barbui C, Gray R, et al. Clinical interventions for treatment non-adherence in psychosis: meta-analysis. Br J Psychiatry 2003; 183:197–206
2. Petersen L, Jeppesen P, Thorup A, et al. A randomised multicentre trial of integrated versus standard treatment for patients with a first episode of psychotic illness. BMJ 2005;331:602
3. Pharoah FM, Rathbone J, Mari JJ, et al. Family intervention for schizophrenia. Cochrane Database Syst Rev 2003;CD000088
4. Zygmunt A, Olfson M, Boyer CA, et al. Interventions to improve medication adherence in schizophrenia. Am J Psychiatry 2002;159:1653–1664
5. Marshall M, Lockwood A. Assertive community treatment for people with severe mental disorders. Cochrane Database Syst Rev 2000; CD001089
6. Grawe RW, Falloon IRH, Widen JH, et al. Two years of continued early treatment for recent-onset schizophrenia: a randomised controlled study. Acta Psychiatr Scand 2006;114:328–336
7. American Psychiatric Association. Diagnostic and Statistical Manual of Mental Disorders, Fourth Edition, Text Revision. Washington, DC: American Psychiatric Association; 2000
8. First MB, Spitzer RL, Gibbon M, et al. Structured Clinical Interview for the Diagnostic and Statistical Manual of Mental Disorders, Fourth Edition, Patient Version. Washington, DC: American Psychiatric Press; 1997
9. Falloon IR. Optimal treatment for psychosis in an international multisite demonstration project. Optimal Treatment Project Collaborators. Psychiatr Serv 1999;50:615–618
10. Falloon IRH, Fadden G. Integrated Mental Health Care. Cambridge, England: Cambridge University Press; 1993
11. Tarrier N, Barrowclough C, Vaughn C, et al. The community management of schizophrenia: a controlled trial of a behavioural intervention with families to reduce relapse. Br J Psychiatry 1988;153:532–542
12. Magana AB, Goldstein JM, Karno M, et al. A brief method for assessing expressed emotion in relatives of psychiatric patients. Psychiatry Res 1986;17:203–212
13. Wearden AJ, Tarrier N, Barrowclough C, et al. A review of expressed emotion research in health care. Clin Psychol Rev 2000;20:633–666
14. Ventura J, Green MF, Shaner A, et al. Training and quality assurance with the Brief Psychiatric Rating Scale: the drift busters. Int J Methods Psychiatr Res 1993;3:221–244
15. Moher D, Schulz KF, Altman D. The CONSORT statement: revised recommendations for improving the quality of reports of parallel-group randomized trials. JAMA 2001;285:1987–1991
16. Kemp R, Hayward P, Applewhaite G, et al. Compliance therapy in psychotic patients: randomised controlled trial. BMJ 1996;312:345–349
17. Kemp R, Kirov G, Everitt B, et al. Randomised controlled trial of compliance therapy: 18-month follow-up. Br J Psychiatry 1998;172:413–419
18. Fernandez RS, Evans V, Griffiths RD, et al. Educational interventions for mental health consumers receiving psychotropic medication: a review of the evidence. Int J Ment Health Nurs 2006;15: 70–80
19. Gray R, Wykes T, Gournay K. From compliance to concordance: a review of the literature of interventions to enhance compliance with antipsychotic medication. Int J Ment Health Nurs 2002;9:277–284
20. Byerly MJ, Fisher R, Carmody T, et al. A trial of compliance therapy in outpatients with schizophrenia or schizoaffective disorder. J Clin Psychiatry 2005;66:997–1001
21. O'Donnell C, Donohoe G, Sharkey L, et al. Compliance therapy: a randomised controlled trial in schizophrenia. BMJ 2003;327:834
22. Kissling W. The current unsatisfactory state of relapse prevention in schizophrenic psychoses: suggestions for improvement. Clin Neuropharmacol 1991;14(suppl 2):S33–S44
23. Weiden P, Rapkin B, Mott T, et al. Rating of Medication Influences (ROMI) scale in schizophrenia. Schizophr Bull 1994;20:297–310
24. Fenton WS, Blyler CR, Heinssen RK. Determinants of medication compliance in schizophrenia: empirical and clinical findings. Schizophr Bull 1997;23:637–651
25. Kamali M, Kelly L, Gervin M, et al. Psychopharmacology: insight and comorbid substance misuse and medication compliance among patients with schizophrenia. Psychiatr Serv 2001;52:161–163, 166

COPYRIGHT 2007 PHYSICIANS POSTGRADUATE PRESS, INC © COPYRIGHT 2007 PHYSICIANS POSTGRADUATE PRESS, INC
